# Priorities for successful use of artificial intelligence by public health organizations: a literature review

**DOI:** 10.1186/s12889-022-14422-z

**Published:** 2022-11-22

**Authors:** Stacey Fisher, Laura C. Rosella

**Affiliations:** 1 Dalla Lana School of Public Health, University of Toronto, Toronto, ON Canada; 2 Public Health Ontario, Toronto, ON Canada; 3 Vector Institute for Artificial Intelligence, Toronto, ON Canada; 4 ICES, Toronto, ON Canada; 5 Institute for Better Health, Trillium Health Partners, Mississauga, ON Canada; 6 Department of Laboratory Medicine and Pathobiology, Temerty Faculty of Medicine, University of Toronto, Toronto, ON Canada

**Keywords:** Artificial intelligence, Public health, Health policy

## Abstract

Artificial intelligence (AI) has the potential to improve public health’s ability to promote the health of all people in all communities. To successfully realize this potential and use AI for public health functions it is important for public health organizations to thoughtfully develop strategies for AI implementation. Six key priorities for successful use of AI technologies by public health organizations are discussed: 1) Contemporary data governance; 2) Investment in modernized data and analytic infrastructure and procedures; 3) Addressing the skills gap in the workforce; 4) Development of strategic collaborative partnerships; 5) Use of good AI practices for transparency and reproducibility, and; 6) Explicit consideration of equity and bias.

**Supplementary Information:**

The online version contains supplementary material available at 10.1186/s12889-022-14422-z.

## Background

Rapid growth in the creation and accessibility of health-related data, together with advances in data storage, computational power, and analytic capacity, has brought about opportunities for artificial intelligence (AI) and its subfields to improve public health. AI can be broadly defined as the imitation of human cognition by a machine [1], or more specifically in this context, software systems that interpret and synthesize data through learning and problem solving to achieve a given goal [2]. The objective of the public health system is to keep people healthy and prevent injury, illness and premature death [3] through six essential functions (Table 1) [4]. Public health organizations responsible for the delivery and oversight of public health are making significant efforts to plan how to incorporate AI into these functions. Notably, however, the considerations for public health are distinctly different from those for clinical or health care systems. In this paper, we identify opportunities and challenges associated with the use of AI for public health and discuss six key priorities for successful implementation of AI by public health organizations.

**Table 1:** Public health functions [4]

1) Health promotion
2) Health surveillance
3) Health protection
4) Population health assessment
5) Disease and injury prevention
6) Emergency prediction, prepardness and response

## Using AI to improve public health

The creation and availability of novel streams of data relevant for health has increased dramatically over the last 20 years, providing detailed insight into social, behavioral, and environmental determinants of health never before possible. For example, data from social media, web search engines and forums, news media, mobile devices and apps provide information about the social determinants of health that is more nuanced than that from traditional sources [5–7]. Wearable devices allow for the collection of detailed information about personal movements and physiological measurements [8]. Environmental sensors collect spatial data about air pollution, water quality, environmental noise, weather conditions, and green space [9]. Much of this data is being produced continuously and can be analyzed in real-time using powerful and increasingly available application programming interfaces (APIs). Furthermore, linkage of these novel data sources to traditional public health data, including that from administrative records, electronic health records, census and health survey data greatly expands potential use. This increase in the type, size and complexity of health-related data has presented new opportunities for public health organizations to use AI methods to improve how they engage in six essential public health functions (Table 1) [4].

Just as AI has been proposed as the cornerstone of precision medicine, it also has the potential to facilitate improved targeting of population health interventions and policy to populations that are most in need. Known as ‘precision public health’, AI can be used to inform how the right intervention can be targeted to the right population at the right time [10]. Health promotion activities can potentially be improved to be better targeted to those that need it most and contribute to system efficiencies using AI technologies [11]. For example, individuals with mixed opinions about hookah tobacco smoking have been identified using sentiment analysis of Twitter data, facilitating public health campaigns targeted at those who may be most receptive [12]. Similar data and natural language processing methods have also been used to identify individuals at risk of suicide [13, 14]. Interactive online tools or apps powered by AI technologies can also provide highly accessible individualized risk assessment and risk reduction recommendations that are more engaging and motivating than traditional approaches, for example, for chronic disease prevention [15] and management [16] or to encourage behavioral change [17]. The World Health Organization recently released Florence, a ‘digital health care assistant’, as part of their AI for Quitting Tobacco initiative (www.who.int/campaigns/Florence) [18]. Using computer-generated imagery, animation and AI, Florence is designed to help people quit tobacco and additionally combat misinformation about COVID-19, through online, face-to-face conversation [19].

Public health surveillance is traditionally performed using population health surveys, clinical data, and public health reporting systems. Access to new data sources and AI methods provides opportunities to identify emerging health threats and develop a more detailed understanding of population disease and risk factor distributions, often with improved geographic resolution. AI-powered approaches can also provide more up-to-date information as data are can be collected, processed, and analyzed in real-time. Public health surveillance dashboards powered by web-accessible news and social media data have been developed to display health events both geographically and temporally [20, 21]. SENTINEL, for example, is a syndromic surveillance tool developed using natural language processing and neural network algorithms, that processes over 1.8 million tweets a day to predict disease occurrence and identify potential outbreaks in real-time [21]. News articles are collected to provide context, and an intuitive user interface displays event predictions geographically and over time compared to weekly counts from the United States Centers for Disease Control. AI can also be used to summarize surveillance information from unstructured sources. For example, natural language processing analysis of free-text information in death certificates has been used to identify potential drug overdose deaths months prior to traditional coding and data release [22]. There is also potential for natural language processing to be used to de-identify personal health information [23].

Health protection and disease and injury prevention can also be improved by AI. Random forest models and k-means clustering has been used to predict bacteria concentration in beach water [24], and natural language processing and web-search engine data have been used to investigate foodborne illness outbreaks [25]. The Chicago Department of Public Health has trained a random forest model to identify children at high risk of lead poisoning and prioritize homes for lead inspections using historical blood lead level tests and home lead inspection data, child characteristics, property value assessments and census data [26]. AI has also been used to select ‘peer change agent’ individuals for a peer-mediated HIV prevention initiative for youth experiencing homelessness, using influence maximization [27].

Population health assessment involves understanding the health of communities, population sub-groups and the determinants of health to improve health policies, services and research to identify effective public health interventions [4]. Gradient boosting decision trees have been used to predict the incidence of preventable hospitalizations for population health planning purposes [28]. A deep learning algorithm has been shown to reduce the spread of tuberculosis in India by informing the use of limited resources [29]. AI can also improve population health management by better identifying population subgroups most in need [30, 31], estimating the potential effects of policy change [32], automatically translating scientific literature [33], and assisting in the production of systematic reviews of public health interventions [34].

AI can also be used for emergency prediction, preparedness, and response. For example, natural language processing and machine learning techniques have been used to detect and track infectious disease outbreaks using commercial flight itineraries, climate data and animal and insect population data for early detection of COVID-19 in Wuhan, China [35] and prediction of international Zika transmission [36].

## Challenges associated with using AI for public health

There are many challenges, risks, and limitations to using AI for public health, including worsening of health inequities (especially in rural, underprivileged communities and in the developing world), poor model interpretability, structural challenges including data sharing, insufficient infrastructure and lack of public health workforce training in AI, and other ethical and privacy concerns.

Although AI has the potential to decrease health inequities through their identification and the subsequent targeting of resources, it also has the potential to create, sustain or exacerbate inequities [37]. This is of particular relevance to public health, given that public health activities are focused at the level of the population, rather than the individual, and therefore AI use for public health activities may influence health inequities more so than its use in other areas [38]. Inequities can arise throughout the AI development pipeline in factors that affect research question selection, representativeness of the data, choice of outcome definition, optimization decisions made during algorithm development, and post-deployment decisions [39]. For example, an algorithm used in hospitals to allocate health care services to patients in the United States was found to be biased against Black patients [40]. Among Black and White patients who were equally sick, Black patients were assigned lower risk scores and were therefore less likely to receive additional services. This bias arose because the algorithm was designed to predict health care costs rather than illness; as Black patients tend to have poorer access to care, they also tend to cost the health care system less. Data representativeness is also a major concern, as AI can learn and amplify biases present in data. For example, a widely-used machine learning technique for text representation known as ‘word embedding’ displays gender stereotypes, such as ‘man is to computer programmer as woman is to homemaker’ or ‘father is to doctor as mother is to nurse’ [41]. More examples of how inequities can occur throughout the AI development pipeline can be found in Chen et al., 2021 [39].

There are also unique AI concerns for rural and underprivileged communities and developing nations. Unequal access to AI-powered technologies (e.g., due to the lack of computational resources, skilled labor, or internet access) can cause health inequities to arise as there is unequal opportunity to benefit [38]. Individuals of higher socioeconomic status also generally benefit from the introduction of innovative health technologies more so than those of lower socioeconomic status, and often adopt these technologies more quickly, which can result in increased social inequality [42, 43]. An evaluation of the role AI may play in achieving the Sustainable Development Goals, most of which are relevant to public health, suggests that AI will influence all 17 goals, with the potential to enable 134 targets and hinder 59 (of a total 169 targets) [44]. For example, AI may support population provision of resources important to public health including food, water and health care, and make more efficient use of limited resources. However, gender equality may be hindered as AI-powered tools may exacerbate discrimination against women and minorities by learning societal biases, inequalities by education may widen as jobs in AI generally require more qualifications, and there are concerns about the huge computational and energy requirements of many AI technologies which may have a large environmental impact [44].

Another commonly discussed limitation of many AI methods is poor explainability, or interpretability, of AI technologies and their outputs. Many AI algorithms are described as a ‘black box’ as models can contain many variables modelled in nonlinear and complex ways, making it difficult or impossible for a human to understand how the output was arrived at. This lack of interpretability can cause skepticism and be detrimental to user trust, especially in the health context [45, 46]. In public health specifically, model interpretability has been recognized as an obstacle to the widespread adoption of AI technologies [47], likely in part due to the population scope of public health action and the risk-averse tendency of government and policymakers. Some machine learning models are viewed as more interpretable than others, including penalized regression methods and single decision trees; however, this perceived advantage often comes at the cost of model performance [48].

Additionally, most traditionally trained public health professionals have not had the training to develop, evaluate or implement AI-based technologies. As such, AI in public health is currently limited and does not take full advantage of the capabilities of the methods or the richness of available data. For example, a recent scoping review of machine learning for population health prediction found that few studies utilized big data, with a median feature size of only 17, and few models used non-traditional sources of health data [49]. It is therefore unsurprising that studies evaluating the use of AI methods for clinical prediction based on relatively limited data sources have found little advantage to using machine learning methods over traditional statistical methods [50]. There are many good resources that can serve as a starting point for public health professionals interested in enhancing AI, machine learning and big data skills [5, 51–54].

The use of AI and big data in public health also raises privacy concerns. Linkage of multiple anonymized data sources is often performed to increase the richness of data prior to analysis with AI techniques; however, this also increases the risk of re-identification of individuals or stigmatization of small groups [53]. Use of data from social media sites, blogs and forums have been associated with risks to individual privacy and autonomy and with the potential for stigmatization [55]. Research in differential privacy aims to mitigate these challenges but remains largely unable to maintain statistical properties that are important for successful AI use [56]. For example, a proposed application of differential privacy to the 2020 United States census was found to radically change population counts for racial/ethnic minorities and lead to biased mortality rate estimates [57].

Structural limitations of AI for public health include difficulties in accessing personal health data and sharing of data across jurisdictions, poor data integration, outdated analytic infrastructure, and lack of software development designed to facilitate the deployment of AI applications into the health system [58, 59].

## How should health organizations incorporate AI into public health activities?

Many health organizations have begun to strategize how to best incorporate AI into their core functions and have developed AI or data-specific strategies, reports, and guidance documents (Table 2; see Additional file 1 for search strategy). Review of these documents reveals many common priorities and approaches. Informed by this review, we have identified six key priorities needed for successful use of AI technologies by public health organizations (Table 3):

1. Contemporary data governance
2. Investment in modernized data and analytic infrastructure and procedures
3. Addressing the skills gap in the workforce
4. Development of strategic collaborative partnerships
5. Use of good AI practices for transparency and reproducibility
6. Explicit consideration of equity and bias

**Table 2:** Data strategies and artificial intelligence guidance reports for health and public health

Country	Organization	Document Title	Link
**Organizational Data Strategies**			
Canada	Statistics Canada	Delivering insight through data for a better Canada (2019) [60]	https://www.statcan.gc.ca/eng/about/datastrategy
Canada	Health Canada	Data strategy (2019) [61]	Not publicly available
Canada	Public Health Agency of Canada	Data strategy (2019) [62]	Not publicly available
Canada	ICES	Envisioning a data science strategy for ICES (2017) [63]	https://www.ices.on.ca/Publications/Atlases-and-Reports/2017/Data-science-strategy
Canada	Public Health Ontario	Informatics strategy (in development) [64]	Not publicly available
United States	National Institutes of Health	Strategic plan for data science (2018) [65]	https://datascience.nih.gov/nih-strategic-plan-data-science
United States	Department of Health and Human Services	HHS Data Strategy: Enhancing the HHS evidence-based portfolio (2018) [66]	https://aspe.hhs.gov/system/files/pdf/261591/2018HHSDataStrategy.pdf
United Kingdom	National Health Services Digital	Data and information strategy (2016) [67]	https://digital.nhs.uk/data-and-information/nhs-digital-data-and-information-strategy#
**AI Reports and Guidance Documents**			
International	World Health Organization	Ethics and governance of artificial intelligence for health: WHO guidance (2021) [68]	https://www.who.int/publications/i/item/9789240029200
International	Pan American Health Organization	Public health in the information society (2017) [69]	https://www.paho.org/salud-en-las-americas-2017/?tag=artificial-intelligence
Canada	Canadian Institute for Advanced Research	Building a learning health system for Canadians: Report of the Artificial Intelligence for Health Task Force (2020) [70]	https://cifar.ca/ai/national-program-of-activities/ai4health-task-force/
Canada	Canadian Institutes of Health Research and the Canadian Institute for Advanced Research	AI for public health equity workshop report (2019) [71]	https://cihrirsc.gc.ca/e/documents/ai_public_health_equity-en.pdf
Canada	Canadian Institutes of Health Research and the Canadian Institute for Advanced Research	Application of artificial intelligence approaches to tackle public health challenges (2018) [72]	https://cihr-irsc.gc.ca/e/documents/artificial_intelligence_approaches-en.pdf
United Kingdom	National Health Service	Artificial intelligence: How to get it right (2019) [73]	https://www.nhsx.nhs.uk/media/documents/NHSX_AI_report.pdf
United Kingdom	Academy of Medical Royal Colleges	Artificial intelligence in health care (2019) [74]	https://www.aomrc.org.uk/wp-content/uploads/2019/01/Artificial_intelligence_in_healthcare_0119.pdf
United States	Center for Open Data Enterprise for the Department of Health and Human Services	Sharing and utilizing health data for AI applications: Roundtable report (2019) [75]	https://www.hhs.gov/sites/default/files/sharing-and-utilizing-health-data-for-ai-applications.pdf
United States	National Academy of Medicine	Artificial intelligence in health care: The hope, the hype, the promise, the peril (2019) [76]	https://nam.edu/wp-content/uploads/2019/12/AI-in-Health-Care-PREPUB-FINAL.pdf
United States	JASON for the United States Department of Health and Human Services	Artificial intelligence for health and health care (2017) [77]	https://www.healthit.gov/sites/default/files/jsr-17-task-002_aiforhealthandhealthcare12122017.pdf

Abbreviations: *AI* artificial intelligence, *HHS* Health and Human Services, *WHO* World Health Organization

**Table 3:** General recommendations to support six strategic priorities for successful use of artificial intelligence by public health organizations

Strategic Priority	General Recommendations
**Governance**	
	- Clarify data leadership roles and responsibilities - Review current organizational governance - Understand and operationalize higher-level governance ○ Involve subject-matter experts in AI, data management and information technology ○ Develop a mechanism for community and public engagement - Establish transparent oversight and accountability
**Infrastructure**	
	- Assess infrastructural and analytic needs - Increase data access - Improve data interoperability - Increase availability of advanced analytic infrastructure and tools ○ Consider investment in distributed data platforms and cloud computing
**Workforce**	
	- Identify and forecast desired skills and competencies, and review existing skills and capacity - Upskill existing staff ○ Increase data literacy across the organization, with a focus on bias and equity considerations - Recruit new staff with desired skills - Engage with trainees; consider development of trainee fellowship programs - Foster multidisciplinary collaboration
**Partnerships**	
	- Identify areas where partnerships may be helpful (e.g., gain expertise, obtain or share access to data or infrastructure, engage a wider variety of perspectives) - Consider partnerships with: ○ Local, provincial/state, federal government ○ Educational institutions ○ Private sector
**Good AI Practices**	
	- Default to transparent data and analytic processes and following reproducible and open science principles whenever possible - Ensure access to and use of practical guidelines for AI development, evaluation, and implementation
**Equity and Bias**	
	- Carefully evaluate and assess potential sources of bias throughout development (including sub-group validation) and implementation - Carefully consider potential biases that may exist in the underlying data used to train models - Consider use of an existing ethical AI framework - Foster diverse AI teams - Engage with the community

### Contemporary data and analytic governance

Every public health organization exists within a larger governance context. Comprehensive understanding of relevant legislation, policies and procedures that govern use of AI for health is therefore integrally important to the safe and successful use of AI for public health activities. This governance exists at different levels, from the international and federal level to organization-specific governance frameworks designed to guide the strategic and efficient management of data and AI technologies. All the organizational documents we reviewed discussed organizational governance and the associated challenges. However, we argue that public health organizations need to focus on understanding and operationalizing higher-level governance rather than reinterpreting into organization-level governance frameworks.

Importantly, this should include the intimate involvement of subject-matter experts in AI, data management and information technology to help ensure that higher-level governance is being interpreted appropriately, operationalized realistically, benefits and risks are both fully understood, and that unnecessary restrictions are not being implemented. It is important to also recognize that the higher-level governance context can change, and that organizational governance must be able to easily adapt. The European Union General Data Protection Regulation represented a massive shift in data protection and privacy and has prompted review of privacy regulation around the world [78]. Canada’s Privacy Act, for example, is currently under review [79].

Organization-level governance should focus on the development and maintenance of effective and efficient data and information technology (IT) systems within the constraints of higher-level regulation. This should include an emphasis on data procurement, linkage and access, privacy, data and IT interoperability, investment in and maintenance of IT infrastructure, prioritization of AI projects, and workforce management of AI, data, and IT personnel. Common governance priorities identified in the documents reviewed include transparent and clear definition of roles and responsibilities and strict oversight and accountability [60–62, 66, 69]. Several organizations have established new roles to lead data governance activities, including a Chief Data Officer at the Public Health Agency of Canada (PHAC)[62] and Health Canada[61], and a Chief Data Strategist at the United States National Institutes of Health [65]. Individuals in these roles are tasked with leading data strategy implementation in collaboration with relevant organizational data councils. Other organizations have prioritized increased communication and coordination between relevant individuals and councils responsible for data governance activities [66].

The World Health Organization proposed a framework for ethics and governance of AI for health which includes: 1) protect autonomy; 2) promote human well-being, human safety and the public interest; 3) ensure transparency, explainability and intelligibility; 4) foster responsibility and accountability; 5) ensure inclusiveness and equity, and; 6) promote artificial intelligence that is responsive and sustainable [68].

Community governance, which involves participation and engagement of the public in decision-making about one’s community, has become recognized as particularly important when considering First Nations’ and Indigenous data and information. The First Nations Principles of OCAP (Ownership, Control, Access, and Possession) were developed to protect Canadian First Nations’ data and information and ensure that it is used and shared in a way that brings benefit to the community while minimizing harm (www.fnigc.ca). [80]. Similarly, the CARE (Collective Benefit, Authority to Control, Responsibility and Ethics) Principles for Indigenous Data Governance are global principles for governance of Indigenous data (www.gida-global.org/care), [81] and EGAP (Engagement, Governance, Access and Protection) is a governance framework for health data collected from Black communities (www.blackhealthequity.ca). [82]

### Investment in modernized data and analytic infrastructure and procedures

Modernization of organizational data infrastructure and procedures is widely recognized by health organizations as vital to moving forward with AI application and strategic use of data [60–67, 70]. A common priority of all organizational strategies we reviewed was to improve data access [60–67] by reducing administrative barriers, reviewing, and revising data use agreements, exploring new data de-identification techniques and establishing remote access to data and analytic tools. Investment in distributed data platforms and cloud computing infrastructure is widely discussed as a means of facilitating rapid and seamless data access in addition to improving data storage and increasing computational power for advanced analytics [60, 62, 63, 65, 66, 70]. These platforms may also reduce infrastructure and maintenance costs in the long-term, compared to local data centers [65]. Health Canada additionally provides access to data through application programming interfaces [61], which Statistics Canada are also looking to use to provide data access to Government of Canada departments [60].

Many organizations are also seeking to improve data interoperability. The NHS is aiming to modernize data infrastructure and increase interoperability through development of a Data Services Platform that will serve as a single place for data collection, processing and management [67]. Similarly, the United States National Institutes of Health (NIH) has goals to connect their data systems and reduce data ‘silos’ [65]. Interoperability is also a primary goal of the Statistics Canada Data Strategy, which they are seeking to improve through the development and use of open data standards [60]. Similarity, Health Canada is aiming to improve data standardization, consolidation and integration through use of open standards and sharing of expertise [61]. Easily accessible data documentation, essential for data interoperability, has also been prioritized in several of the organizational data strategies we reviewed. Examples of this include the Health Canada Information Reference Model [61], the United States NIH Data Discovery Index [65] and a data holding inventory by PHAC [62]. Some organizations are also seeking to improve data interoperability through use of common data models, schema for data harmonization and standardization [61, 63, 65]. Use of existing commercial tools, technologies and services as opposed to internal development of project or organization-specific data infrastructure is also recognized as a means of improving system interoperability and data integration both within and outside of an organization [60, 61, 72]. Increased data linkage is also a common organizational priority [63–67].

In addition to modern data infrastructure and procedures, successful use of AI also requires advanced analytic infrastructure and tools. Many organizational strategies outline plans to increase organizational capacity for advanced analytics by assessing organizational needs [61], increasing computational power [60, 62], facilitating access to new analytic tools [60–65, 67], and through pilot projects using AI methods [62, 63]. It is important to establish what analytic tools are needed to enable AI use, as most traditional public health tools are incapable and/or are not familiar to those with AI or machine learning expertise. For example, Python (Python Software Foundation) [83] and R (R Foundation for Statistical Computing) [84] are programming languages commonly used to develop machine learning models, and Git is a popular, free, and open-source version control system that tracks coding changes. TensorFlow, which is also free and open-source, is an end-to-end software library for machine learning that is especially effective at efficiently deploying machine learning algorithms [85]. Public health professionals do not traditionally use these tools currently, so those with expertise in computer science, AI, and maching learning must determine the appropriate infrastructure, software and tools needed to perform advanced analytics. Several organizations also recognized the importance of flexibility in accessing new analytic tools to enable ‘nimble and agile data analytics’ [60–62].

### Addressing the skills gap in the workforce

Workforce training is an important step in facilitating AI adoption [86, 87]. Successful use of big data, advanced analytic methods and AI requires a workforce with strong data literacy and capacity in data management, statistics, computer science, software engineering, data privacy, bias, and ethics, among other skills. All organizational data strategies we reviewed recognized the importance of building a workforce that is educated in these skills and outlined plans to achieve it through training staff and leveraging existing skills, targeted recruitment, and engagement with trainees and educational institutions. Most of the strategies also discussed the intention to increase organizational skills and capacity in AI specifically [60–66].

Upskilling existing staff will generally be an important priority of all public health organizations interested in increasing use of AI. It should first involve identifying and forecasting desired data and analytic competencies and a review of existing organizational skills and capacity [62, 64, 67]. Data literacy, defined as the ability to collect, manage, evaluate, and critically apply data [88], is widely recognized as a vital competency to be emphasized across health organizations interested in AI [60–62, 64, 66]. Statistics Canada has developed data literacy training products including the Framework for Responsible Machine Learning Processes at Statistics Canada [89] and introductory training videos on machine learning, data stewardship and data quality, among others [90]. The Government of Canada developed a Digital Academy in 2018 to “help federal public servants gain the knowledge, skills and mindsets they need in the digital age”, and includes training on data literacy and competencies, cloud computing, cyber security, AI and machine learning, among other topics [91]. The Digital Academy is being used by PHAC and Health Canada to train existing and new employees [61, 62]. PHAC outlined many additional training strategies, including use of third-party web-based tools, self-directed learning, trainings customized to specific audiences, development of a Data 101 onboarding package and specific training in innovation [62]. The United States Department of Health and Human Services (HHS) is looking to increase data science and statistical training opportunities and increase multidisciplinary collaboration across the organization, recognizing that informed data science decisions require a wide range of skills and expertise [66]. The NHS is seeking to leverage existing skills through the creation of teams specializing in particular data skills and through external and internal staff rotation, in addition to the development of training programs [67]. ICES is looking to develop a data science staff education strategy, which will include data science workshops and increased exposure of analysts and methodologists to the R statistical programming language [63]. Statistics Canada is seeking to develop a culture of ‘continuous learning’ [60]. Continuous learning can be facilitated in part by increased access to scientific publications, a priority of PHAC [62].

Targeted recruitment of new employees is another means of developing a workforce educated in data science and AI and is an important component of the workforce development plan for many health organizations [60–62, 64–67]. As individuals with many of the desired skills have not traditionally worked in health, it is important to consider how to best attract and retain this talent. This begins with increasing data literacy across the organization and provision of appropriate infrastructure and tools, and is further facilitated by an organizational culture that is receptive to change and taking risks. A goal of the Health Canada Data Strategy, for example, is to provide employees with “an agile collaborative space for learning and innovative uses of data” and is seeking to create a strong data culture and environment that values experimentation and learning from failure [61]. PHAC and PHO have similar innovation and risk-taking goals [62, 64]. The United States HHS strategy outlines four approaches to hiring data scientists, including participating in job fairs and industry events, creation of intern and fellowship programs, hiring of individuals with non-traditional backgrounds into senior positions and making use of existing specialized hiring programs [66]. The United States NIH is looking to develop a Data Fellows program, in which individuals with desired skills are recruited from the private sector and academia for short-term national service sabbaticals [65].

It has been suggested that trainees are “the glue that tie researchers together”, fostering interdisciplinary research and learning [71]. Engagement with trainees increases awareness of organizational data science career possibilities and provides the organization with access to new and developing data science talent. Most of the organizational strategies we reviewed include engagement with trainees and educational institutions as part of their workforce development plans [60, 62, 63, 65, 66]. In Canada, the Health System Impact Fellowship [92], funded by the Canadian Institutes for Health Research, has an equitable AI stream in which PhD and postdoctoral fellows with skills in computer science, AI and data science are embedded within health system organizations to help solve critical health system challenges [93]. Both PHAC and PHO have hosted fellows through this program. Other organizations have plans for similar organization-specific fellowship programs, including Statistics Canada [60] and the United States NIH and HHS [65, 66].

### Development of strategic collaborative partnerships

Development of collaborative partnerships is an important component of strategic data use and successful AI implementation. Collaboration can come in many forms and be used to gain expertise, obtain or share access to data and infrastructure, and engage a wider variety of perspectives. The CIFAR AI for Public Health Equity report recommends collaboration of public health professionals and researchers with computer science and AI researchers, in addition to a wide range of other groups (e.g., sociologists, political scientists, engineers, civil society and citizen scientists, people with lived experience, policymakers) to help ensure health equity when using AI technologies [71]. ICES is looking to facilitate development and implementation of data and computational infrastructure through partnerships in addition to continued collaboration with external scientists and research institutions for data science and AI expertise [63]. Many governmental organizations plan to closely collaborate with other local, provincial/state or federal government organizations or departments, sharing infrastructure, data and expertise [60–62, 65, 66]. As mentioned previously, engagement with trainees can be greatly beneficial to workforce development and promotes collaboration with educational institutions. Collaboration with the private sector can additionally be advantageous. The United States NIH, for example, is seeking to leverage private sector infrastructure through strategic collaboration [65].

### Use of good AI practices for transparency and reproducibility

Transparency is intended to foster trust and prevent harm and is one of the most common ethical AI principles [94]. Transparency in AI often refers to efforts to increase explainability and interpretability and generally involves detailed disclosure of how an AI model or technology was developed, how it performs, the data it uses, how it is deployed and used, discussion of limitations, and may involve sharing of source code and data [94]. Transparent AI promotes freedom and autonomy by increasing the public’s knowledge of AI and promoting informed consent [94]. Transparency does not only necessitate sharing of source code or data, which may be proprietary; however, a minimal level of transparency should be expected if an AI technology is to be used, especially at the population level for public health purposes. In particular, the transparency in how the model was created and decision-points of model development and validation should be made available. The EQUATOR (Enhancing the QUAlity and Transparency Of health Research) Network is an international initiative promoting transparent reporting of health research literature by encouraging wider use of robust reporting guidelines (www.equator-network.org). Particularly relevant to AI is the ‘Transparent Reporting of a multivariable prediction model for Individual Prognosis or Diagnosis’ (TRIPOD) reporting guideline [95], with an upcoming extension specifically for models developed using machine learning methods [96]. Health Canada is looking to improve data transparency and access to data as a means of increasing public confidence in decision-making [61]. The NHS has committed to greater transparency in data use and in algorithmic development and deployment [97]. The WHO has identified transparency, explainability and intelligibility are one of the core ethical principles of AI use for health [68].

While there will be claims of commercial concerns for proprietary intellectual property and even controversial concerns over “research parasites” [98], some minimal level of transparency is necessary. Before clinical acceptance of systems can be expected, peer-reviewed publication of model performance and sources of training data should be expected just as much as population descriptions in randomized controlled trials. This is necessary to clarify the representativeness of any models and what populations to which they can reasonably be expected to apply.

Closely related to the principle of transparency is open science. Open science is a movement to make scientific research transparent and accessible to all. Open science reduces research waste, facilitates reproducibility, and allows for AI to more easily benefit everyone (related to the ethical principle of beneficence). One of the main recommendations of the CIFAR Public Health Equity report [71] and the NHSX [73] is that organizations should use and allow for sharing of datasets, data repositories, and resources. Open science efforts include providing the public with access to data and information, use of open data standards and open-source programs, open-source code, use of open data, and open access publication. A commitment to increased data sharing was stated in several organizational strategies [61, 62, 65, 66] Several organizations also prioritized increasing their use of open data standards to improve interoperability [60, 62, 65]. Statistics Canada is committed to increased transparency of data use and processes, including through publishing of code on the Open Data Portal [60]. The Pan American Health Organization also lists open science and open data as guiding principles [69]. Principles of transparency and open science, however, need to be carefully balanced with privacy and confidentiality through organizational governance. Protections must be in place to ensure data protection and security and prevent discrimination of individuals and small population sub-groups [99]. The Health Canada data strategy states that “getting privacy and ethics right will actually enable increased use and sharing of data, since data stewards will have knowledge of the data limits and have confidence that they can use and share data without harm.” [61]

There also exist practical guidelines for developing and reporting prediction models. The detailed explanation and elaboration document for the previously mentioned TRIPOD reporting guideline lists many practical recommendations for developing well performing models, including predictor measurement and description, defining the outcome, handling of missing data and variable preprocessing [100]. We anticipate that the upcoming TRIPOD-Machine Learning guideline will be especially useful [96]. Other practical considerations for the use of AI in health include the importance of representative data, cross-validation and data leakage, overfitting, and rigorous model evaluation [101, 102].

### Explicit consideration of equity and bias

Health equity has been defined to mean that “all people can reach their full health potential and should not be disadvantaged from attaining it because of their race, ethnicity, gender, age, social class, language and minority status, socio-economic status, or other socially determined circumstances” [103]. Ethical considerations exist at all stages of the AI development and implementation pipeline, from problem selection and data collection to post-deployment [39]. As public health professionals are trained to think about bias, generalizability and equity, they are especially able to recognize and inform mitigation strategies for AI use in public health in collaboration with computer science and AI professionals.

Best practices have been established to guide the development, implementation, and evaluation of AI-powered technologies to ensure that they are not only useful, but also do not create, sustain, or exacerbate health inequities. Many principles and frameworks have been developed to guide the ethical use of AI [94]. The United Kingdom (UK) National Health Service (NHS) [67, 73] and UK National Academy of Medical Royal Colleges [74] recommends following the ‘Guide to Good Practice for Digital and Data-Driven Health Technologies’ [97]. This document developed by the UK Government outlines ten principles to guide the development and implementation of data-driven health and care technologies. Many organizations [61, 65, 104] refer to the FAIR data principles: research data should be finable, accessible, interoperable and reusable [105]. A report from the United States National Academy of Medicine refers to several existing frameworks and principles including ‘Artificial Intelligence at Google: Our Principles’ [106] and the ‘AI Now Report 2018’ [107]. The WHO has produced a guidance document with six principles to promote the ethical use of AI for health [68]. Common principles among many of these frameworks include transparency, non-maleficence, responsibility and accountability, privacy, freedom and autonomy, beneficence, trust and justice, fairness and equity [94].

It is important to carefully assess potential sources of bias during model development and deployment. This should involve careful consideration of the data used to train the model as well as validation of model performance across population sub-groups such as race, sex, or geography. A practical guide to assessing organizational algorithms for bias is the ‘Algorithmic Bias Playbook’ [108]. Written for organizational executives, technical teams, as well as policymakers and regulators, this step-by-step resource walks through how to screen organizational algorithms for bias, retrain biased algorithms, and prevent future bias.

For ethical AI use, the public should be engaged and informed about how their data is used, how AI applications may influence their lives, and be given space to voice their preferences and concerns [38, 70, 76, 94]. The CIFAR Artificial Intelligence for Health (AI4H) task force report recommends that “members of the public and patients should be included as active partners in the development, governance and evaluation of AI4H policies and strategies” [70]. For public health specifically, it has been recommended that rural and remote communities and people with lived experiences be engaged in relevant AI research and implementation from project initiation [71, 72]. Another means of engaging with the public is through citizen science, in which members of the public lead or participate in scientific research, recommended by two reports from the United States [76, 77]. The United States NIH is committed to facilitating citizen science in their Strategic Plan for Data Science, through public access to data, tools and education in addition to exploration of other community engagement models [65].

Lastly, scientific teams greatly benefit from being diverse and multidisciplinary [39, 71, 76]. An AI report from the United States National Academy of Medicine recommends that AI teams are diverse in “gender, culture, race, age, ability, ethnicity, sexual orientation, socioeconomic status, privilege, etc.” to promote the development of impactful and equitable AI tools [76]. It has been argued that there is a direct link between lack of diversity in the AI workforce and discriminatory AI tools; AI tools can not only be biased technically, but also be biased by the people and the context in which they are built, in a ‘discrimination feedback loop’ [109]. Bias can arise from unrepresentative or biased data, but also in why and how a system was designed, how it was constructed and the metrics by which its success or failure is assessed [39]. Given the wide reach of public health and its focus on health equity, diversity is particularly important to ensure the development of equitable AI tools for public health. The United States NIH is looking to increase workforce diversity, in part through their Big Data to Knowledge Diversity Initiative [65]. The United States HHS has goals to promote multidisciplinary data science teams and increase cross-program and interdepartmental collaboration [66].

## Moving towards an AI-enabled public health organization

**Fig. 1:**
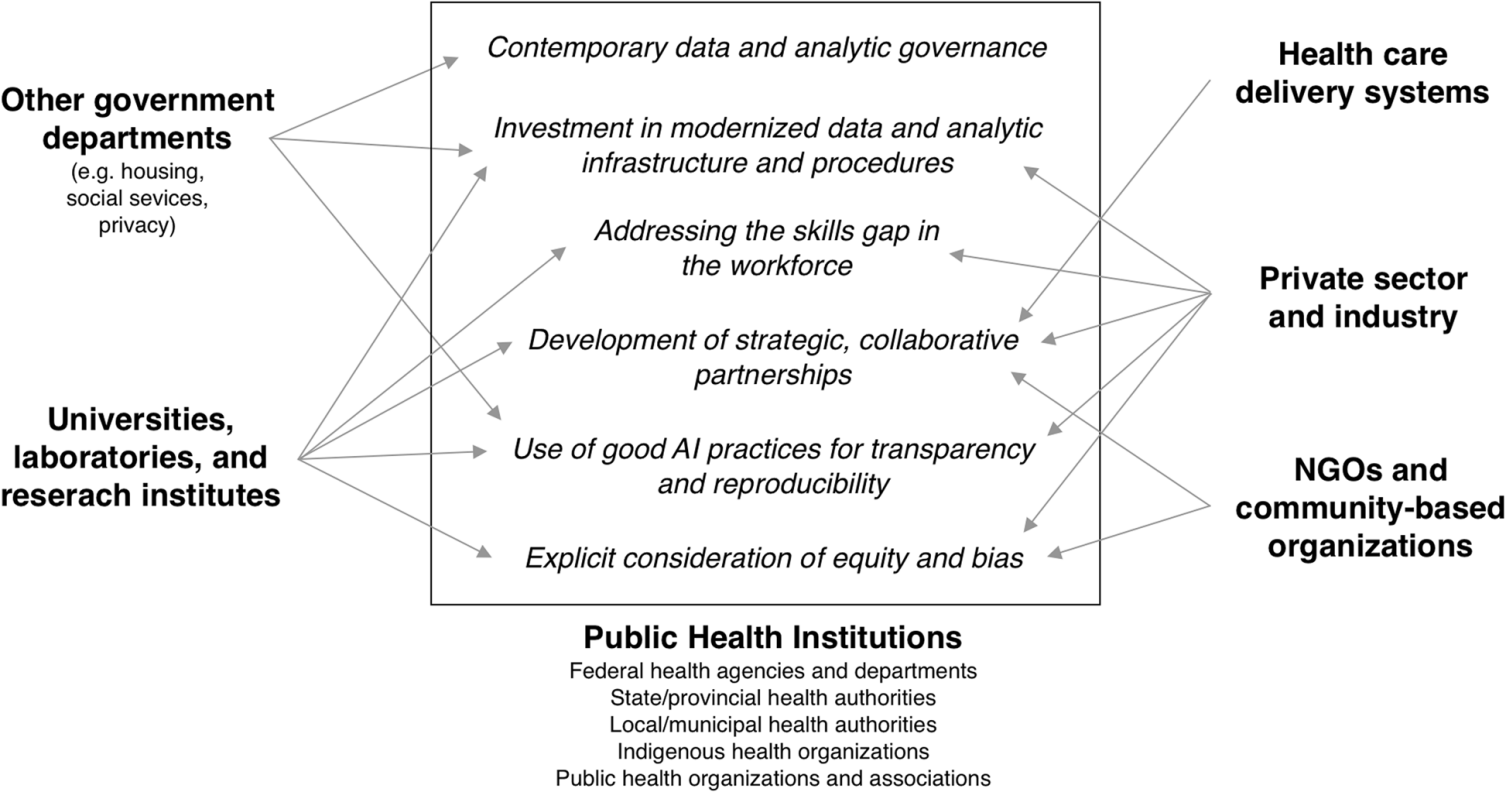
Six key priorities for successful use of artificial intelligence by public health organizations and their relationship to the broader public health system

Among those that provided a timeline, organizations generally planned to take steps toward all identified priorities in parallel [60, 62, 67] although progress on governance issues and infrastructure are likely needed before significant progress can be made on other priorities. Figure 1 lists the six key priorities and their relationship to the broader public health system.

Initial governance activities should include clarification of data and analytic leadership roles and responsibilities, and a review of current organizational governance to ensure alignment within the larger governance context. Existing data and analytic infrastructure should be evaluated in consultation with data management, data science and AI experts to identify priorities for modernization and identify places where a small early investment may have a large impact. An early focus on data standardization and documentation may have long-term benefits to data interoperability within the organization. Pilot projects evaluating use of new infrastructure and/or advanced analytic methods should be initiated in several application areas. For example, the United States NIH piloted use of a cloud computing environment with a small number of test datasets to establish the architecture, policies and processes for storage, sharing and analysis of data through the NIH Data Commons Pilot [65].

To begin to address the skills gap and establish a workforce educated in data and analytic skills, desired skills and competencies should be identified and forecasted, and existing skills and capacity reviewed to inform the development of employee training and targeted hiring programs. Training of existing and new employees at all levels of seniority in data literacy, bias and equity should be prioritized early, as organizational culture changes slowly. Areas where partnerships may be beneficial should be identified and relationship-building prioritized. Organizations should consider use of an existing ethical AI framework to guide AI activities and default to transparent data and analytic processes and following open science principles whenever possible. Access to practical guidelines for AI development, evaluation, and implementation should be ensured.

## Conclusions

To successfully realize the potential for AI to improve public health it is important for public health organizations to thoughtfully develop strategies for AI implementation. This should include review and modernization of exiting organizational data and analytic governance and infrastructure, addressing the AI and data science skills gap, development of strategic collaborative partnerships, and use of AI best practices including explicit consideration of equity.

## Supplementary Information

**Additional file 1.**

## Data Availability

All data analyzed in this study are included in the published article.

## Abbreviations

AI: Artificial intelligence
AI4H: Artificial Intelligence for Health
API: Application programming interfaces
CARE: Collective Benefit, Authority to Control, Responsibility and Ethics
CIFAR: Canadian Institute for Advanced Research
CIHR: Canadian Institutes of Health Research
EGAP: Engagement, Governance, Access and Protection
EQUATOR: Enhancing the QUAlity and Transparency Of health Research
HHS: Health and Human Services
IT: Information technology
NHS: National Health Service
NIH: National Institutes of Health
OCAP: Ownership, Control, Access, and Possession
PHAC: Public Health Agency of Canada
PHO: Public Health Ontario
TRIPOD: Transparent Reporting of a multivariable prediction model for Individual Prognosis or Diagnosis
UK: United Kingdom
WHO: World Health Organization
