# Supplementary material for: Priorities for successful use of artificial intelligence by public health organizations: a literature review
Source: BMC Public Health. 2022 Nov 22;22:2146. doi: 10.1186/s12889-022-14422-z (PMC9682716; doi:10.1186/s12889-022-14422-z)
Supplement: Supplementary file 1 — Additional file 1. [file 12889_2022_14422_MOESM1_ESM.docx]

**Artificial intelligence for public health: Priorities for successful use by public health organizations**

Stacey Fisher and Laura C Rosella

**Appendix**

Search Strategy

An environmental scan of organizational strategies, reports and guidance documents focused on the use of artificial intelligence and machine learning for public health was performed. Documents with a data science focus were also included if the use of data for artificial intelligence or machine learning purposes was discussed. Documents with a clinical or general health focus were included if public health was also mentioned. Online literature was scanned for publicly available documents in March 2020. Keywords used in the search were “artificial intelligence”, “machine learning”, “data science”, “informatics”, “public health”, “population health”, “strategy”, “report”, “framework”, “initiative” and “action plan”. Additional documents were identified through discussions with public health organizations.
